# Antivax attitude in the general population along the autism-schizophrenia continuum and the impact of socio-demographic factors

**DOI:** 10.3389/fpsyg.2023.1059676

**Published:** 2023-04-21

**Authors:** Luca Tarasi, Sara Borgomaneri, Vincenzo Romei

**Affiliations:** ^1^Dipartimento di Psicologia, Università di Bologna and Centro studi e ricerche in Neuroscienze Cognitive, Università di Bologna, Cesena, Italy; ^2^IRCCS Fondazione Santa Lucia, Rome, Italy

**Keywords:** vaccinal attitudes, schizotypal traits, autistic traits, cognitive styles, education, age, ASD-SSD continuum

## Abstract

**Introduction:**

One of the most important inventions in human history is vaccines. However, to date a consistent amount of people exhibit a hesitant approach toward them and mixed results have emerged in the attempt to characterize which factors may play a role in predicting such negative attitude. Here, we aimed at investigating how the individual scoring along the autism-schizophrenic continuum component and socio-cultural factors contribute toward vaccination attitudes in the general population.

**Methods:**

To test whether individual position along the autism-schizophrenic continuum could predict vaccine attitude, we used principal component analysis (PCA) to extract the component showing diametric loading between the Schizotypal Personality Questionnaire (SPQ) and Autistic Quotient (AQ) subscales. Then, we performed a series of multiple linear regression analyses to understand the relation between the ASD-SSD continuum component and Vax scores. We also included socio-demographic factors (i.e., gender, education level, and age) as predictors.

**Results:**

Multiple regression analysis revealed that the closer the individual lied on the positive schizotypal pole, the higher was their negative attitude toward vaccines. A diametric, more favorable disposition was found for individuals closer to the autistic end of the continuum. Furthermore, we reported that among the socio-cultural factors, only age can be considered a significant predictor of vaccination attitudes, with younger participants showing a more positive attitudes toward vaccination, while the level of education is an important protective factor in mitigating the negative impact that the proximity to the SSD pole and age play against vaccination disposition.

**Discussion:**

These findings are relevant to improve targeted public health interventions, highlighting the crucial role of demographic, psychological, and social correlates in predicting anti-vax beliefs, which have the devasting potential to increase the spread of infectious disease.

## 1. Introduction

The development of vaccines has represented one of the most important innovations in the history of humanity and medicine, helping to prevent an estimated 3.5–5 million deaths each year (World Health Organisation, 2022). The relevance of vaccination became even more tangible with the emergence of the COVID-19 pandemic as the world’s attention focused on the development of a new vaccine capable of contrasting this virus. Despite the unquestionable value of vaccines in counteracting the spread and reducing the number of hospitalizations and deaths due to virus-related infectious diseases (Bernal et al., 2021), a skeptical attitude toward vaccines and vaccination campaigns implemented by governments around the world is widespread in the general population (Freeman et al., 2020). In this regard, the concept of “hesitancy” has been defined as the behavior of “delaying acceptance or refusal of vaccines despite the availability of vaccine services” (SAGE Working Group on Vaccine Hesitancy, 2014) and, recently, it has been included in the top-10 threats to global health (World Health Organisation, 2019). This hesitant approach has important practical repercussions at both individual (e.g., increased risk of suffering lethal effects from the virus) and socio-economic level (e.g., increased spending by health care systems) (Mullooly et al., 1994; Tenforde et al., 2021). In recent years, there are numerous lines of research investigating what factors determine attitudes toward vaccination (Yaqub et al., 2014). Research in this area is prompted by the fact that they represent serious risk factors regarding infectious diseases, as a negative attitude has been linked to actual vaccine uptake (Latkin et al., 2022). Socio-cultural factors, such as gender, education level and age appear to play a role in explaining vaccination attitudes (Bertoncello et al., 2020). However, the directionality of the effects is not fully understood due to the presence of mixed findings. For example, Chen et al. (2021) highlighted that the level of education was negatively associated with intention to vaccinate, whereas lower education levels were associated with vaccine hesitancy (Robertson et al., 2021) and higher conspiracy beliefs about COVID-19 (De Coninck et al., 2021) in other studies. Regarding gender, higher vaccine hesitancy rates (Morales et al., 2022) and lower vaccine intention (Zintel et al., 2022) have been reported in women compared to men, but opposite results are also reported. For example, in Latin America, the Caribbean, and high-income countries, women reported being less hesitant than men (Flor et al., 2022). Regarding the influence of age, some studies have highlighted that vaccine hesitancy was higher in younger age groups (Robertson et al., 2021) but others pointed in opposite direction showing that age was positively associated to vaccine hesitancy (Moscardino et al., 2022; Ouyang et al., 2022).

These mixed results could be due to the fact that vaccine attitude differs across time, country, and vaccine type. Thus, a variable predicting a particular vaccination attitude in a given context could cease to make a difference when contingencies change. For example, a worldwide survey demonstrated that the European region is the least confident toward vaccine safety, while Bangladesh, Ecuador, and Iran reported highest agreement that vaccines are important (Larson et al., 2016). Moreover, socio-demographic variables may impact only specific dimensions of vaccine attitude. For example, education increases confidence in the importance and efficacy of the vaccine, but not safety (Larson et al., 2016).

In addition to socioeconomic and cultural elements, psychological factors such as personality traits, and beliefs appear to direct attitudes toward vaccines (Hornsey et al., 2018). Among these factors, a critical element in dictating vaccinal attitude could be the type of predictive style adopted by the individual. According to Bayesian brain theories, our perception/decision arises from an integration-like mechanism between externally derived signal (sensory evidence) ascending the cortical hierarchy in a bottom-up flow and signal derived from internal model (prior belief) that descends the cortical hierarchy in a top-down flow. The balanced integration of these two sources of information would lead to adaptive choice and behavior (Tarasi et al., 2022a). Crucially, there are clinical/sub-clinical manifestations in which the decision-making process leans overly toward priors or sensory evidence. For example, the autism-schizophrenia continuum model (Tarasi et al., 2022b) posits that autism spectrum disorders (ASD) and schizophrenia spectrum disorders (SSD) would be associated with a different weight assigned to top-down and bottom-up information (Tarasi et al., 2021; Andersen, 2022; Ursino et al., 2022), resulting in behavioral/cognitive patterns pointing in opposite directions. Specifically, whereas prior information would be overweighted in positive SSD, input-based information would be the core upon which ASD relies in decision making. The evidence for this diametricality is manifold: positive schizotypy (which is characterized by the presence of ideas of reference, magical/bizarre thinking and unusual perceptual experiences) tends to be associated to belief-driven perception (Schmack et al., 2013), less deliberate decision-making processes (Leer et al., 2015) and resistance toward evidence that goes against pre-established beliefs (Buchy et al., 2007), whereas people with high autistic-like traits are characterized by overweighting of external evidence (Van de Cruys et al., 2014), detail-oriented processing approach (Alink and Charest, 2020) and deliberate and logical thinking style (Brosnan et al., 2016; Lewton et al., 2019). Importantly, these peculiarities in the predictive style that characterize positive SSD and ASD could subserve a different disposition toward vaccines. Since higher levels of positive schizotypy and delusion proneness have been associated to anti-scientific beliefs (i.e., telepathy; Raine, 1991), to conspiracy theory about COVID-19 (Larsen et al., 2021; Acar et al., 2022), to prior-driven perception (Schmack et al., 2013) and individuals with psychotic disorders tend to refuse COVID-19 vaccination more than the general population (Hassan et al., 2022), we hypothesize that individuals lying toward the positive SSD pole of the continuum may show a general negative attitude toward vaccines. Furthermore, it is possible to speculate that the propensity for deliberate processing and the tendency to gather much evidence before making a decision observed in ASD may be related to a positive attitude toward vaccination, as the adoption of a deliberative and analytical thinking style increases the inclination to reject COVID-19 conspiracy theories/theorists (Swami and Barron, 2021). Evidence in support of this hypothesis comes from populations with clinical autism that show higher odds of being vaccinated for COVID-19 (Weinstein et al., 2021; Shea et al., 2022). However, the adoption of a deliberative cognitive style devoted to information gathering (Brosnan et al., 2014; Quinde-Zlibut et al., 2020) neither eliminates the possibility of developing erroneous beliefs (Georgiou et al., 2021) nor automatically implies a positive approach to vaccination as this also depends, for example, on the type of source and information channels from which the evidence on which the attitude rests is drawn.

To shed light on these hypotheses, the present study aims to explore, for the first time, whether positive schizotypal and autistic traits predispose to a particular attitude toward vaccination as measured by the Vaccination Attitudes Examination (VAX) Scale (Martin and Petrie, 2017), by assuming that being closer to the positive schizotypal vs. autistic side of the continuum could favor a negative vs. positive attitude toward vaccination. In the study we will also consider the possible role that gender, education level, and age play in these relationships given their highlighted role in the literature in moderating vaccination attitudes.

## 2. Materials and methods

### 2.1. Participants

268 (Female = 186) individuals within the general population took part in the study by completing an online form. All participants signed a written informed consent prior to taking part in the study, which was conducted in accordance with the Declaration of Helsinki and approved by the Bioethics Committee of the University of Bologna. Participants provided their demographic data, such as age, gender, and education. The age of the sample ranges from 18 to 76 (*M* = 27.13, SD = 11.67) while participants’ years of education range from 13 to 25 (*M* = 20.06, SD = 2.32).

### 2.2. Measures

#### 2.2.1. Schizotypal traits

Subclinical traits associated with the SSD were measured with the Schizotypal Personality Questionnaire (SPQ; Raine, 1991). This self-report questionnaire is composed of 74 questions divided in 9 subscales (ideas of reference, magical thinking, social anxiety, unusual perceptual experiences, constricted affect, no close friends, odd behavior, odd speech and suspiciousness) that can be further organized in 3 main factors (cognitive-perceptual, interpersonal and disorganization) in which participants are asked to answer questions regarding different aspects of their personality, behavioral preferences and cognitive styles, in addition to questions concerning sensorial experiences and beliefs, with “Yes” or “No” statements. We used the original scoring methods, assigned the response a binary code (no = 0; yes = 1).

#### 2.2.2. Autistic traits

We used the Autism-Spectrum Quotient test (AQ; Baron-Cohen et al., 2001). This self-report questionnaire is composed of 50 questions divided in 5 subscales in which participants are asked, similarly to the SPQ, to answer questions regarding different aspects of their personality, behavioral preferences, cognitive styles, as well as potential discomfort connected to specific sensorial experiences. Each AQ subscale is composed by 10 items addresses a psychological feature present in ASD: imagination (assesses imaginative ability), communication (assessing the weakness in communication skills), social skills (assessing the presence of poor social skills), attention to detail (assessing the exceptional attention to detail), and attention switching (assessing poor attention switching ability/strong focus of attention). The sum of the scores obtained in each subscale provides a global score, with higher values indicating higher levels of autistic traits. We used the original scoring methods, converting each item into a dichotomous response (agree/disagree) and assigned the response a binary code (0/1). Descriptive statistics of the SPQ and AQ self-report and their intercorrelations are included in the supplementary materials (Supplementary Table S1; Supplementary Figure S1).

#### 2.2.3. Vaccination attitude

We used the 12-item Vaccination Attitudes Examination (VAX) scale (Martin and Petrie, 2017) to measures vaccine attitude. This self-report demonstrated good internal consistency, convergent validity, and construct validity (Wood et al., 2019). VAX is composed by four subscales (3 items each): mistrust of vaccine benefit (Vax-mistrust), worries about unforeseen future effects (Vax-worries), concerns about commercial profiteering (Vax-prof), and preference for natural immunity (Vax-natur). Responses are assigned by judging the degree of agreement with the 12 statements using a 6-level Likert scale (from “Strongly Agree” to “Strongly Disagree”). A higher score indicates more negative attitudes toward vaccinations.

### 2.3. Statistical analysis

#### 2.3.1. Autism-schizophrenic continuum

To identify the autism-schizophrenia axis, a principal component analysis (PCA) was performed on the correlation matrix of the AQ and SPQ subscales. To verify the adequacy of the dataset for the proposed analysis, the Kaiser–Mayer–Olkin (KMO) measure and the Bartlett’s test were used. The first two principal components were extracted and we selected the second one which, according to previous literature (Dinsdale et al., 2013; Del Giudice et al., 2014; Zhou et al., 2019; Nenadić et al., 2021), is supposed to capture the diametric relationship between these two conditions, as it is inversely loaded with schizotypal and autistic subscales.

#### 2.3.2. Multiple regression analysis

We performed a series of multiple linear regression analyzes (using the enter method) to understand the relation between the ASD-SSD continuum factor and demographical data with Vax scores. Therefore, we placed the total Vax score or Vax subscales as the dependent variable and entered the PC2 scores and demographic indices as predictors. We also investigated possible multicollinearity issues.

## 3. Results

### 3.1. Analyses were conducted using SPSS 26 and RStudio v2021

#### 3.1.1. Principal component 2 tracks the diametric dimension between ASD and SSD

To test whether individual position along the ASD-SSD axis could predicted vaccine attitude, we used principal component analysis (PCA) to extract the component showing diametric loading between the SPQ and AQ subscales. Bartlett’s (*p* < 0.01) test and the Kaiser-Meyer-Olkin (KMO = 0.88) proved the adequacy of the data for the proposed analysis. According to previous literature, the second component condenses the dimensions that show a diametric pattern whereas the first component condenses the common features between these two personality traits. Our results pointed in this direction (Supplementary Figure S2): the two components accounted for 53.5% of the variance, with the first dimension showing positive loadings with all subscales of the SPQ and AQ, especially the subscales indicating socio-communicative dysfunction. Conversely, the second component exhibited both negative and positive loadings with the two questionnaires. Specifically, the positive dimensions of the SPQ (ideas of reference, magical thinking, and unusual perceptual experience) loaded positively on the second component, while all subscales of the AQ (except attention to detail) loaded negatively. Crucially, negative schizotypal subscales pointed in the same direction of the AQ dimensions. These psychometric results corroborate previous findings showing the presence of comparable cognitive and perceptual phenomena that tie these two dimensions together (Trevisan et al., 2020). This result confirmed the presence of a diametrical structure, with autistic traits and positive schizotypal traits placed at the opposite ends of a single continuum.

#### 3.1.2. The position along the ASD-SSD continuum and age predicted vaccinal attitude

**Figure 1:**
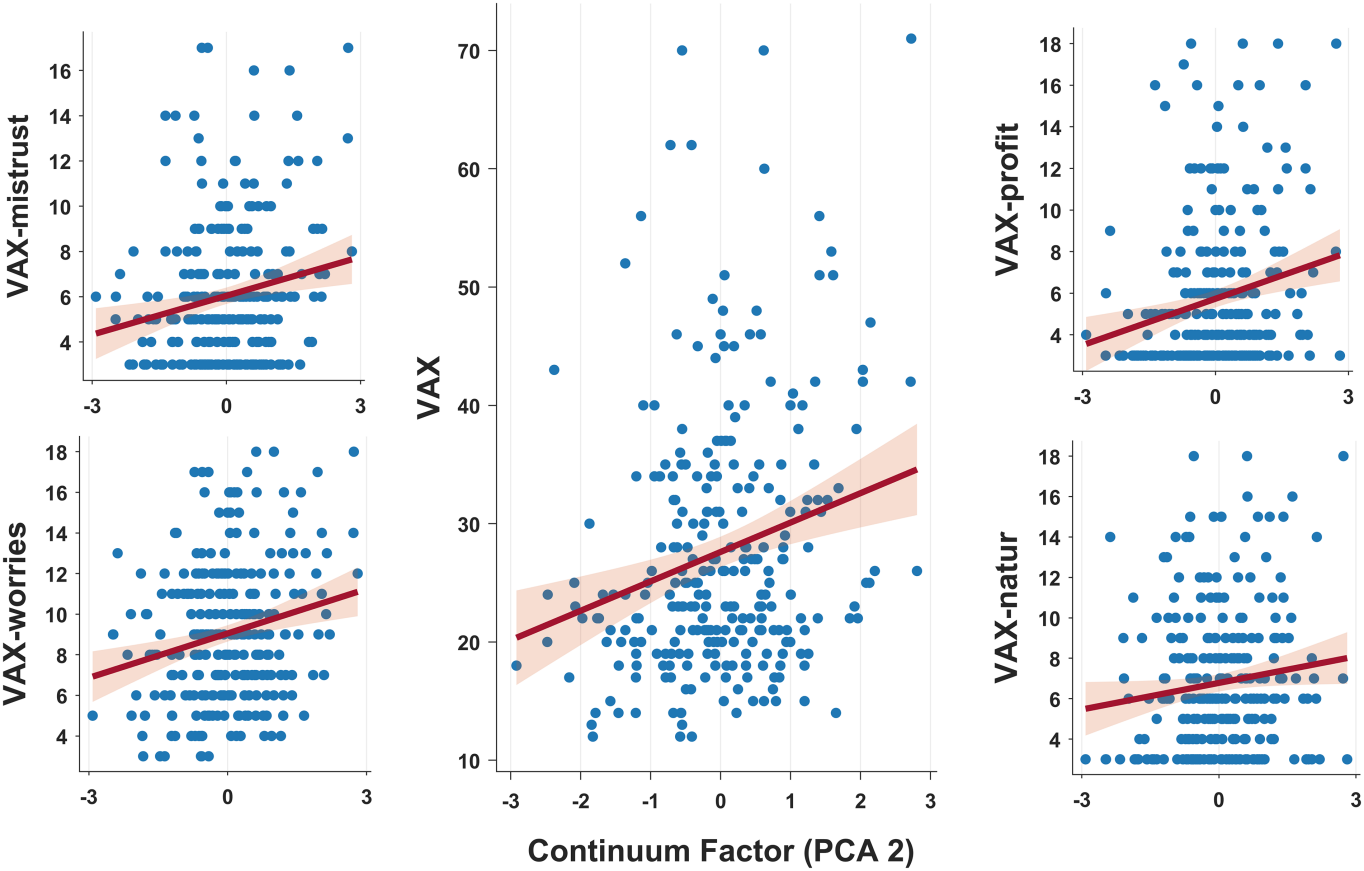
Graphical representation of the relationship between individuals’ scores on the ASD-SSD continuum factor (PC2, *x*-axis) and attitudes toward vaccination. Multiple regressions conducted showed that proximity to the positive schizotypal (vs. autistic) pole promoted widespread negative (vs. positive) attitudes toward vaccination.

We conducted multiple regression analyzes to investigate whether the ASD-SSD continuum factor (Figure 1) and/or socio-demographic factors could predict vaccination attitude. We found no multicollinearity problems in the multiple regressions conducted (max VIF value: 1.014). The analysis (Table 1) showed that:

1. In the first regression, we entered Vax total score as the dependent variable. Results showed that the regression was significant (*F*_4, 263_ = 5.52, *p* < 0.01, *R*^2^ = 0.08). As can be seen in Table 1, both the ASD-SSD continuum factor and Age emerged as significant predictors of a general negative attitude toward vaccination.
2. In the second regression, we considered as dependent variable the subscale Vax-mistrust. The regression was again significant (*F*_4, 263_ = 2.44, *p* = 0.047, *R*^2^ = 0.04) but only the ASD-SSD continuum factor emerged as significant predictor of increased distrusting of vaccine benefit.
3. In the third regression, we considered as dependent variable the subscale Vax-prof. The regression was again significant (*F*_4, 263_ = 5.11, *p* < 0.01, Adj. *R*^2^ = 0.07) with both the ASD-SSD continuum factor and Age predicting a greater tendency to have concerns about commercial profits related to vaccines. In addition, a trend very close to the statistical threshold emerged that pointed to a reduced tendency to associate vaccine spread as being motivated by economic rather than public health concerns as educational levels rise.
4. In the fourth regression, we considered as dependent variable the subscale Vax-worries. The regression was again significant (*F*_4, 263_ = 8.44, *p* < 0.01, *R*^2^ = 0.11). Again, the ASD-SSD continuum factor and Age emerged as predictors of increased worry about vaccine-related future adverse events.
5. In the fifth regression, we considered as dependent variable the subscale Vax-natur. The regression showed only a trend toward statistical significance (*F*_4, 263_ = 1.82, *p* = 0.12, *R*^2^ = 0.03), but the ASD-SSD continuum factor significantly predicted a preference for natural exposure to viruses rather than through vaccination practices.

**Table 1:** Regression coefficients of the ASD-SSD component and socio-demographic variables on vaccination attitudes.

	Vax		Vax-Mistrust		Vax-worries		Vax-profit		Vax-natural	
	*β*	*p*	*β*	*p*	*β*	*p*	*β*	*p*	*β*	*p*
ASD-SSD continuum factor	**0.23**	**0.00**	**0.19**	**0.00**	**0.22**	**0.00**	**0.21**	**0.00**	**0.12**	**0.04**
Education	−0.06	0.34	0.01	0.80	−0.04	0.52	−0.11	0.07	−0.05	0.44
Gender	0.03	0.60	0.02	0.70	0.08	0.15	0.02	0.70	−0.03	0.66
Age	**0.16**	**0.01**	0.00	0.99	**0.25**	**0.00**	**0.14**	**0.02**	0.10	0.10

*β*, Standardized beta*; p*, *p*-value.Two predictors were significantly associated with individual attitudes toward vaccination. The first is the ASD-SSD continuum factor, which is a significant predictor for all Vax subscales and for overall negative attitudes toward vaccination. The second predictor is age, which was found to be related to an overall adverse attitude toward vaccination, greater fear of long-term harm, and concerns about commercial gains associated with vaccine spread.Significant predictors are represented in bold.

All in all, the regressions carried out showed that there are two main predictors that succeed in intercepting individual attitudes toward vaccination. The first and most important is the ASD-SSD continuum factor, which is a significant predictor for all Vax subscales and for a general negative attitudes toward vaccination. In addition, we determined that the magical thinking subscale appears to be the dimension along the continuum most strongly associated with a negative disposition toward vaccination (Supplementary Table S3). The second main predictor was age, which was found to be related to a general negative attitude toward vaccination, increased fear of long-term harms, and concerns about commercial gains related to vaccine dissemination.

#### 3.1.3. Education levels moderate the relationship between the ASD-SSD continuum factor, age, and vaccination attitude

**Figure 2:**
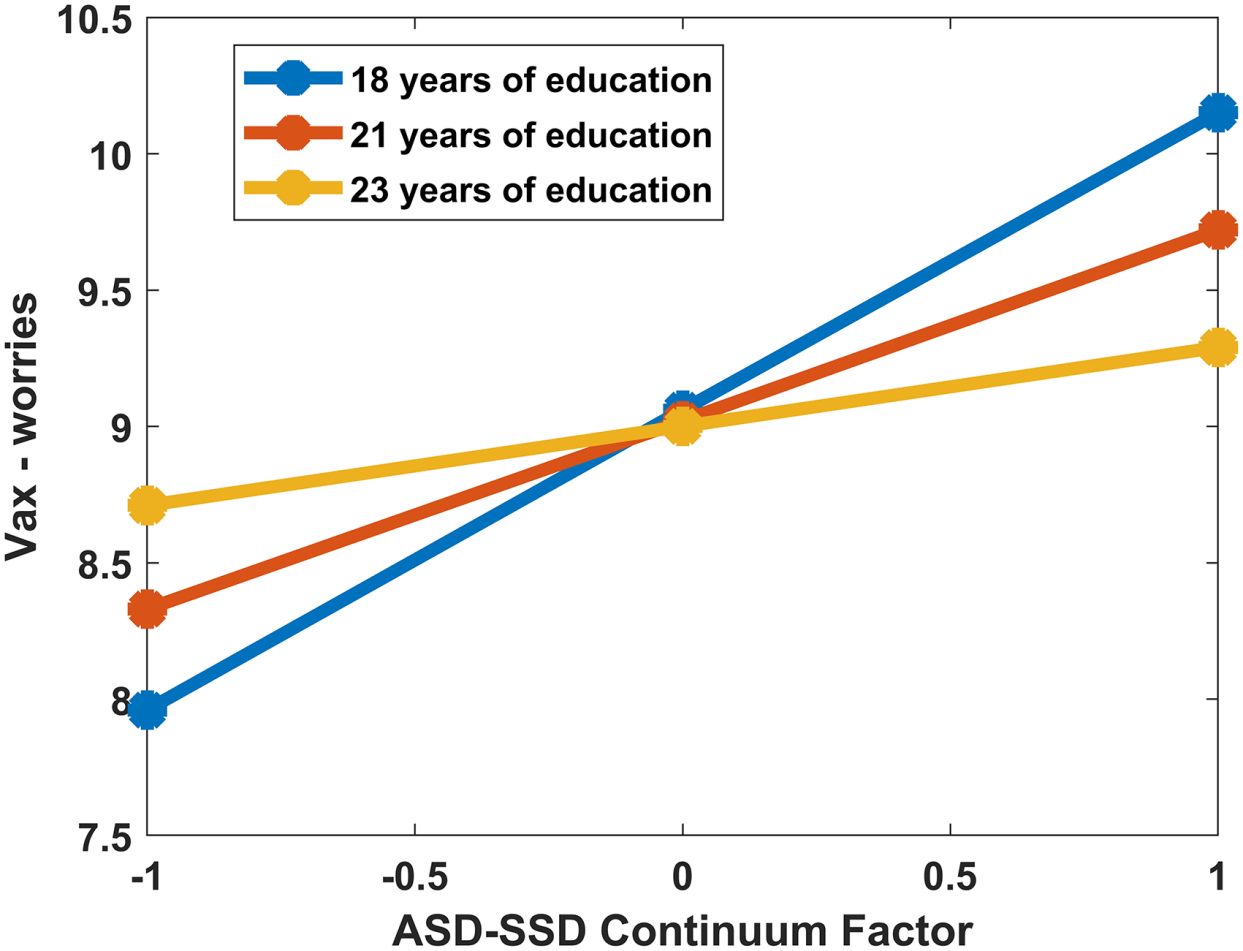
From the graph, it is possible to visualize how the education level factor interacts with the ASD-SSD continuum factor in explaining attitudes toward vaccination. In particular, the ASD-SSD continuum factor is related to increased concern about vaccine-related adverse events in a different way depending on education level. Specifically, higher levels of education mitigate the effect that the proximity to the SSD pole exerts on Vax-worries subscale scores.

Having assessed the relationship between the ASD-SSD continuum factor, age, and negative attitudes toward vaccination, we have explored, using a moderator analysis conducted in RStudio, whether contextual variables related to the level of education attained could have a protective effect, dampening the effect that the proximity to the SSD pole and age have on attitudes toward vaccination. The analyzes were carried out by mean-centering the predictors and calculating 95% confidence interval (CI) based on 2000 bootstrap iterations and assessing whether it overlapped with the zero value. The conducted analyzes disclosed:

1. The presence of a significant interaction between the ASD-SSD continuum factor and education level considering the Vax-worries subscale (*b* = − 0.17, 95% CI = [− 0.355; − 0.003]); therefore, PC2 scores correlated with a concern about vaccine-related adverse events in a different fashion according to education level (Figure 2). Specifically, higher levels of education dampen the effect that the proximity to the SSD end of the continuum has on scores in the Vax-worries subscale.
2. The presence of a significant interaction between age and education level considering the Vax-worries subscale (*b* = − 0.014, 95% CI = [− 0.028; − 0.003]); thus, lower levels of education magnify the impact that age has on Vax-worries subscale.
3. The presence of a significant interaction term between age and education level considering the Vax-profit subscale (*b* = − 0.019, 95% CI = [− 0.028; − 0.008]); again, level of education appears to have a protective effect on vaccine attitude, as a high level is associated with a dampening of the positive association between age and concerns about vaccine-related profits. It should be noted that both moderation analyses are equally significant when removing individuals who are still in educational age.

## 4. Discussion

Vaccine hesitancy has been designated as one of the threats to global health by the World Health Organization and has been a strikingly salient issue during the COVID-19 pandemic. This work sought to shed light on the factors that lead to negative attitudes toward vaccines in an adult population. In particular, our study investigated how socio-demographic characteristics and personality traits dictate disposition toward vaccination.

First, we proved that the placement of individuals along the ASD-SSD continuum affected vaccine attitudes. Specifically, the closer the individual was to the schizotypal axis (positive scores in PC2) the more negative attitudes toward vaccines were displayed, whereas disposition was more favorable in participants closer to the autistic end of the continuum (negative scores in PC2). This relationship was statistically significant in all dimensions probed by the Vax questionnaire, demonstrating that position along the ASD-SSD axis pervasively shapes vaccine disposition.

It is noteworthy that not all schizotypal subscales loaded positively with principal component 2 (Supplementary Figure S2), but only the subscales measuring positive symptoms. Positive schizotypal symptoms are genetically related to schizophrenia (Saarinen et al., 2022) and are featured by a number of unusual tendencies and experiences such as odd beliefs, ideas of reference and delusions (Lenzenweger, 2006) which typically does not misrepresent reality as much as in psychosis, but are configured as a sub-threshold psychotic form (American Psychiatric Association, 2013). These unusual sets of thoughts, such as having faith in telepathy, sixth sense, and paranormal phenomena often conflict with mainstream beliefs (Raine, 1991). Thus, the proximity to the SSD pole of the continuum could cause a negative bias against vaccination because of the tendency to escape the culturally provided worldview (e.g., the scientific perspective) and turn to non-traditional channels of information (e.g., conspiracy websites). This could, in turn, lead to the generation of conspiracy-like beliefs about vaccines that fuel a hostile attitude. Crucially, scoring on measures of belief in conspiracy theories are positively predicted by individual schizotypal traits (Darwin et al., 2011), and adoption of a conspiracy perspective on coronavirus is associated with hesitancy toward the COVID-19 vaccine (Allington et al., 2021) and with decreased institutional trust, reduced support for government regulations and physical distancing measures (Pummerer et al., 2022).

Following a Bayesian perspective, these idiosyncrasies manifesting in the SSD pole of the continuum may result from an imbalance in the weight placed on belief-driven (overweighted) and sensory evidence-driven (underweighted) information (Tarasi et al., 2022b). Higher level of delusion-proneness was positively related to a tendency to gather insufficient evidence when forming beliefs (Leer et al., 2015), to a predisposition to establish meaningful patterns where there are none present (i.e., apophenia; Blain et al., 2020) and to manifest a bias against disconfirmatory evidence (Woodward et al., 2007). Therefore, positive SSD features could promote the formation of non-evidence-based beliefs, which would subsequently be overweighted in the decision-making process, as testified by studies showing that a greater tendency for delusional beliefs is associated with prior-driven decision-making (Schmack et al., 2013). At the neural level, the over-weighting of prior information relative to disconfirmed sensory evidence could stem from an imprecision of bottom-up signaling (Tarasi et al., 2022b). For instance, in both schizophrenia and schizotypy, a reduction in the speed of oscillations in the alpha band (IAF) has been consistently observed (Fuggetta et al., 2014; Murphy and Öngür, 2019; Ramsay et al., 2021; Trajkovic et al., 2021; Ippolito et al., 2022). Individual Alpha Frequency is a crucial marker involved in the precise encoding of incoming evidence (Samaha and Postle, 2015; Coldea et al., 2022; Di Gregorio et al., 2022), segregation of sensory information (Cecere et al., 2015; Ronconi et al., 2018; Wutz et al., 2018; Cooke et al., 2019; Sharp et al., 2022) and shown to be associated with long integration windows between sensory modalities leading to high proneness in crossmodal illusory perception (Haß et al., 2017; Ferri et al., 2018; Fenner et al., 2020; Fotia et al., 2021). Crucially, Ramsay et al. (2021) showed that SSD individuals exhibited reduced alpha speed compared to the control group and that this reduction was predictive of lower sensitivity in a visual task and correlated with impaired global cognition. Thus, it is possible that an imprecision in the input signal processing triggers cascade mechanisms that would lead the top-down flow (carrying the belief information) to prevail by directing the activity of the lower brain areas (Schmack et al., 2013; Dzafic et al., 2021; Tso et al., 2021).

Moreover, the study has shown that proximity to the ASD pole of the continuum fosters a positive attitude toward vaccines. This pattern might depend on the fact that the ASD pole is characterized by the tendency to systematization (Baron-Cohen et al., 2003), and hyperponderance of external inputs (Brock, 2012; Karvelis et al., 2018) that would be processed in a more analytical and deliberative way (Brosnan et al., 2016; Lewton et al., 2019). Follow-up studies will be useful to examine whether this positive attitude is actually underpinned by the assumption of a more analytic thinking style as one approaches the autistic end of the continuum.

Looking at socio-demographic variables, only age was found to be a significant predictor of vaccination attitudes. In our sample, young age was shown to be associated with more positive attitudes toward vaccination. This finding is particularly surprising considering that virus infections tend to have potentially problematic outcomes as age increases. Indeed, previous studies have found that older people are more likely to report that they would take a vaccine relative to the younger categories (Lazarus et al., 2021) which instead showed higher levels of hesitancy in the UK population (Allington et al., 2021). However, it should be noted that increasing age heightens susceptibility to misinformation. For example, age is a strong predictor of online fake news dissemination, with users over the age of 65 sharing nearly seven times as many articles from fake news domains as the younger age group (Guess et al., 2019). Therefore, it is conceivable that the enormous amount of false information proliferated about vaccines in the recent years has an increasingly detrimental effect on vaccinal attitudes as age increases.

In addition, although there is some evidence of greater vaccine hesitancy in women than men (Morales et al., 2022), no gender-dependent difference emerged in our sample. However, the one small trend that emerged points to an increased fear of future vaccine-related adverse events in the female gender. This nonsignificant relationship would not be surprising because women are more likely to express concerns about vaccine safety, which may also explain the trend toward higher vaccination rates in male (Bish et al., 2011).

Finally, our exploratory analysis showed that level of education plays a protective role, mitigating the negative effect that being close to the schizotypal axis and age have on attitudes toward vaccination. In fact, with lower levels of education, both proximity to the SSD pole of the continuum and age predict extremely negative attitudes toward vaccination, while with high values of education this relationship is more nuanced. Specifically, education level attenuates the relationship between ASD-SSD continuum factor scores, age and the concern about vaccine-related adverse events (*Vax-worr*) and the relationship between age and concerns about vaccine-related profits (*Vax-prof*). Older people or individuals closer to the SSD pole tend to assume an association between vaccines and hidden risks. This relationship could be brought into play because of conspiracy-type thinking, which links vaccines to risks that manufacturers do not declare. A higher level of education might make individuals more prone to engage in more elaborate, nuanced reasoning and more ready to cast doubt on the validity of their beliefs. In addition, individuals with higher education tend to be more analytically oriented compared with the students with lower level of education (Aarnio and Lindeman, 2005) and this might make it easier for them to seek evidence-based information that counteract the belief about vaccine-related risk, e.g., evidence about the rigid safety steps and protocols that vaccines must prove to have passed before being put on the market. This finding emphasizes that the level of education is an important protective factor that succeeds in mitigating the effect that psychological or demographic variables have on a maladaptive attitude such as a negative vaccination disposition, resulting in benefits at the individual (e.g., reduced risk of adverse events) and social (e.g., reduced cost of care) level.

One limitation of the study concerns the generalizability of the data. Although the online form used for data collection allowed us to reach people from different socio-demographic background, the data sample analyzed has an average age of around 27 years old and an average level of education of 20 years old. This testifies to the fact that the majority of participants are young and educated adults. These limitations can be overcome in future studies by employing differentiated data collection methods and/or a larger sample size. In addition, data were collected on individuals within the general population. Follow-up studies should assess whether a diametric pattern of vaccine attitudes might also emerge by considering individuals at the extreme ends of the continuum (patients with schizophrenia and autism).

In summary, the proposed study revealed that anti-vax attitudes may be favored by proximity to the schizotypal pole of the ASD-SSD continuum, whereas being close to the autistic pole promotes the adoption of a more positive perspective toward vaccination. In addition, demographic factors such as age play a role in shaping vaccination attitudes. Finally, we highlighted the protective role played by education in mitigating the negative impact that schizotypal traits and age have toward vaccines. These findings offer a new understanding of the factors that motivate people to want to reject the science on vaccinations. Understanding these underlying motivations opens up new opportunities in terms of promoting resilient strategies against antivax attitudes by improving tailored public health interventions to reduce the risk deriving from maladaptive attitudes toward vaccines.

## Data availability statement

The raw data supporting the conclusions of this article will be made available by the authors, without undue reservation.

## Ethics statement

The studies involving human participants were reviewed and approved by Bioethics Committee of the University of Bologna. The participants provided their written informed consent to participate in this study.

## Author contributions

VR, LT, and SB contributed to conception and design of the study. LT collected the data, organized the database, and performed the statistical analysis. LT and VR wrote the first draft of the manuscript. SB wrote sections of the manuscript. All authors contributed to manuscript revision, read, and approved the submitted version.

## Funding

VR was supported by Bial Foundation (204/18).

## Conflict of interest

The authors declare that the research was conducted in the absence of any commercial or financial relationships that could be construed as a potential conflict of interest.

## Publisher’s note

All claims expressed in this article are solely those of the authors and do not necessarily represent those of their affiliated organizations, or those of the publisher, the editors and the reviewers. Any product that may be evaluated in this article, or claim that may be made by its manufacturer, is not guaranteed or endorsed by the publisher.
