# Supplementary material for: Antivax attitude in the general population along the autism-schizophrenia continuum and the impact of socio-demographic factors
Source: Front Psychol. 2023 Apr 21;14:1059676. doi: 10.3389/fpsyg.2023.1059676 (PMC10161933; doi:10.3389/fpsyg.2023.1059676)
Supplement: Supplementary file 1 [file Data_Sheet_1.docx]

| **Descriptive Statistics** | | | |
| --- | --- | --- | --- |
|  | **Mean** | **Std. Deviation** | **Cronbach alpha values** |
| AQ | 17.75 | 6.58 | 0.78 |
| AQ-im | 3.51 | 1.81 | 0.39 |
| AQ-soc | 3.10 | 2.06 | 0.59 |
| AQ-as | 4.40 | 2.15 | 0.58 |
| AQ-ad | 4.27 | 2.26 | 0.64 |
| AQ-co | 2.47 | 2.02 | 0.63 |
| SPQ | 20.96 | 13.29 | 0.94 |
| SPQ-ref | 2.40 | 2.38 | 0.79 |
| SPQ-mag | 1.17 | 1.42 | 0.61 |
| SPQ-sa | 3.92 | 2.60 | 0.83 |
| SPQ-perc | 1.77 | 1.84 | 0.70 |
| SPQ-ob | 1.61 | 1.97 | 0.82 |
| SPQ-nf | 1.97 | 2.12 | 0.77 |
| SPQ-os | 3.18 | 2.63 | 0.81 |
| SPQ-aff | 2.21 | 1.89 | 0.71 |
| SPQ-susp | 2.73 | 2.44 | 0.83 |
| VAX | 27.61 | 11.01 | 0.91 |
| VAX mistrust | 6.04 | 3.06 | 0.89 |
| VAX worries | 9.05 | 3.42 | 0.79 |
| VAX prof | 5.74 | 3.57 | 0.90 |
| VAX natur | 6.78 | 3.58 | 0.88 |

Supplementary Materials

Table S1

*AQ = Autistic Quotient; AQ-im = Imagination; AQ-soc = Social Skills; AQ-as = Attention Switching; AQ-ad = Attention to Details; AQ-co = Communication; SPQ = Schizotypal Personality Questionnaire; SPQ-ref = Ideas of Reference; SPQ-mag = Magical Thinking; SPQ-sa = Social Anxiety; SPQ-perc = Unusual Perceptual Experience.; SPQ-ob = Odd Behaviour; SPQ-nf = No Close Friends; SPQ-os = Odd Speech; SPQ-aff = Constricted Affect; SPQ-sus = Suspiciousness.


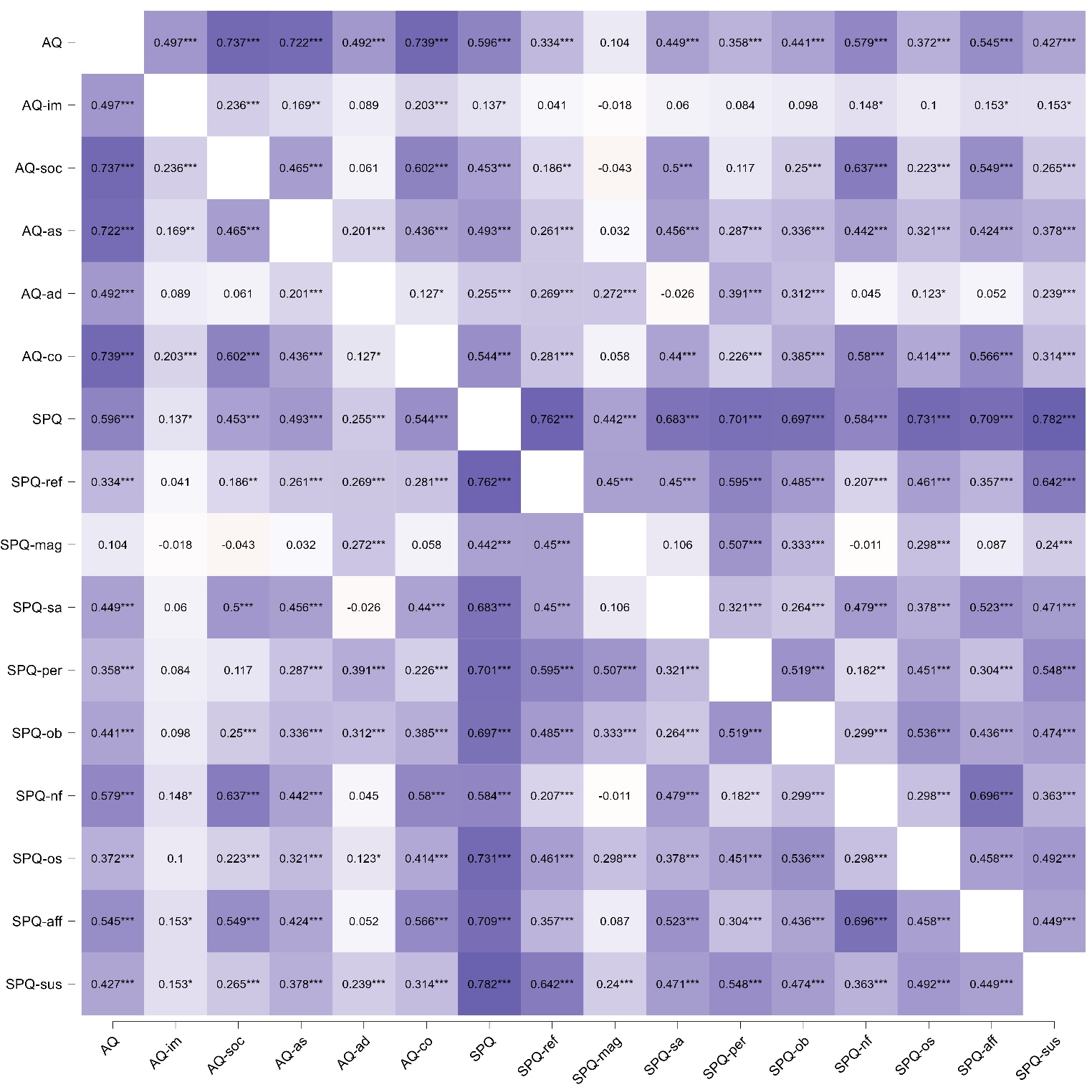


Figure S1. Correlation Coefficient between AQ and SPQ subscales.

*AQ = Autistic Quotient; AQ-im = Imagination; AQ-soc = Social Skills; AQ-as = Attention Switching; AQ-ad = Attention to Details; AQ-co = Communication; SPQ = Schizotypal Personality Questionnaire; SPQ-ref = Ideas of Reference; SPQ-mag = Magical Thinking; SPQ-sa = Social Anxiety; SPQ-perc = Unusual Perceptual Experience.; SPQ-ob = Odd Behaviour; SPQ-nf = No Close Friends; SPQ-os = Odd Speech; SPQ-aff = Constricted Affect; SPQ-sus = Suspiciousness.


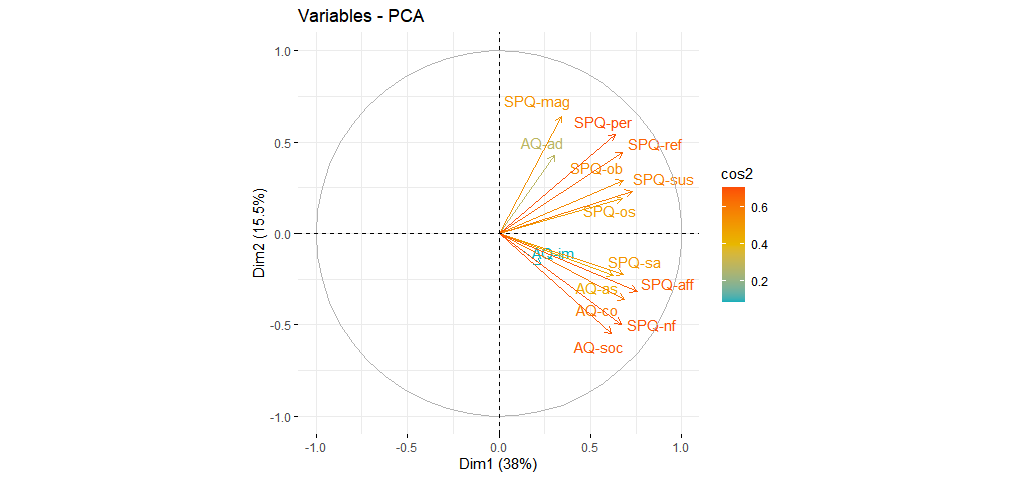


Figure S2. Spatial position of the AQ and SPQ subscales on the two extracted components.

The first component explained 38% of the variance, while the second component explained 15.5% of the variance

* AQ-im = Imagination; AQ-soc = Social Skills; AQ-as = Attention Switching; AQ-ad = Attention to Details; AQ-co = Communication; SPQ-ref = Ideas of Reference; SPQ-mag = Magical Thinking; SPQ-sa = Social Anxiety; SPQ-perc = Unusual Perceptual Experience.; SPQ-ob = Odd Behaviour; SPQ-nf = No Close Friends; SPQ-os = Odd Speech; SPQ-aff = Constricted Affect; SPQ-sus = Suspiciousness.

| **Component Loadings** | | | | | | |
| --- | --- | --- | --- | --- | --- | --- |
|  | | **PC1** | | **PC2** | |  |
| **AQ-soc** |  | 0.617 |  | -0.548 |  |  |
| **AQ-as** |  | 0.627 |  | -0.231 |  |  |
| **AQ-ad** |  | 0.305 |  | 0.429 |  |  |
| **AQ-im** |  | 0.231 |  | -0.172 |  |  |
| **AQ-co** |  | 0.689 |  | -0.362 |  |  |
| **SPQ-ref** |  | 0.677 |  | 0.444 |  |  |
| **SPQ-mag** |  | 0.342 |  | 0.642 |  |  |
| **SPQ-sa** |  | 0.682 |  | -0.223 |  |  |
| **SPQ-per** |  | 0.637 |  | 0.544 |  |  |
| **SPQ-ob** |  | 0.681 |  | 0.292 |  |  |
| **SPQ-nf** |  | 0.671 |  | -0.495 |  |  |
| **SPQ-os** |  | 0.675 |  | 0.192 |  |  |
| **SPQ-aff** |  | 0.757 |  | -0.315 |  |  |
| **SPQ-sus** |  | 0.730 |  | 0.232 |  |  |
|  | | | | | | |

Table S2

Loading of each subscale on the two components.

* AQ-im = Imagination; AQ-soc = Social Skills; AQ-as = Attention Switching; AQ-ad = Attention to Details; AQ-co = Communication; SPQ-ref = Ideas of Reference; SPQ-mag = Magical Thinking; SPQ-sa = Social Anxiety; SPQ-perc = Unusual Perceptual Experience.; SPQ-ob = Odd Behaviour; SPQ-nf = No Close Friends; SPQ-os = Odd Speech; SPQ-aff = Constricted Affect; SPQ-sus = Suspiciousness.

|  | Vax | | Vax-Mistrust | | Vax-  worries | | Vax-  profit | | Vax-  natural | |
| --- | --- | --- | --- | --- | --- | --- | --- | --- | --- | --- |
|  | β | *p* | β | *p.* | β | *p.* | β | *p.* | β | *p.* |
| **AQ-im** | 0.01 | 0.86 | -0.03 | 0.66 | -0.03 | 0.59 | 0.09 | 0.14 | 0.00 | 0.99 |
| **AQ-soc** | 0.07 | 0.36 | 0.11 | 0.16 | 0.11 | 0.16 | -0.05 | 0.53 | 0.06 | 0.43 |
| **AQ-as** | -0.12 | 0.10 | -0.12 | 0.10 | -0.08 | 0.24 | -0.09 | 0.22 | -0.09 | 0.19 |
| **AQ-ad** | 0.09 | 0.15 | 0.03 | 0.60 | 0.12 | 0.06 | 0.08 | 0.19 | 0.05 | 0.41 |
| **AQ-co** | 0.02 | 0.80 | -0.04 | 0.62 | -0.02 | 0.83 | 0.11 | 0.13 | 0.00 | 0.93 |
| **SPQ-ref** | -0.04 | 0.62 | -0.10 | 0.21 | 0.02 | 0.85 | 0.00 | 1.00 | -0.05 | 0.54 |
| **SPQ-mag** | **0.34** | **0.00** | **0.24** | **0.24** | **0.29** | **0.00** | **0.31** | **0.00** | **0.26** | **0.00** |
| **SPQ-per** | -0.02 | 0.78 | 0.09 | 0.26 | -0.05 | 0.51 | -0.43 | 0.60 | -0.05 | 0.52 |

Table S3

The exploratory regressions carried out showed that Magical Thinking was a significant predictor for all Vax subscales and for general negative attitudes toward vaccination. Regarding AQ subscales, a trend pointing in the direction of better attitudes toward vaccination as a function of autistic traits emerges considering the general positive attitudes toward vaccination and the increased trust in vaccination efficacy (AQ-mistrust) as the attention subscale score (AQ-switch) increases. These results pointed in the same direction even when controlling for the age variable.

* AQ-im = Imagination; AQ-soc = Social Skills; AQ-as = Attention Switching; AQ-ad = Attention to Details; AQ-co = Communication; SPQ-ref = Ideas of Reference; SPQ-mag = Magical Thinking; SPQ-perc = Unusual Perceptual Experience.

****** β = Standardized beta; p = p-value.
